# Basketball talent identification: a systematic review and meta-analysis of the anthropometric, physiological and physical performance factors

**DOI:** 10.3389/fspor.2023.1264872

**Published:** 2023-11-14

**Authors:** Miaoyu Han, Miguel-Angel Gómez-Ruano, Alberto Lorenzo Calvo, Jorge Lorenzo Calvo

**Affiliations:** Facultad de Ciencias de la Actividad Física y del Deporte, Universidad Politécnica de Madrid, Madrid, Spain

**Keywords:** basketball, talent identification, anthropometric, physiology, physical performance

## Abstract

**Background:**

The purpose of this study was to do a systematic review and meta-analysis about the anthropometric, physiological, and physical performance that discriminate the physical talent in basketball.

**Methods:**

The search was conducted using the most relevant databases as Web of Science, PubMed, SPORTDiscus and Scopus, according to the PRISMA (2020) guideline. Keywords such as “basketball”, “talented identification”, “anthropometric”, “physiology” and “physical performance” were used for the search, in English and following the “PICOS” question model. Eighteen articles' overall methodological quality was assessed using the Critical Review Forms.

**Results:**

The study found in basketball, height, body mass, Yo-Yo test, *T*-test, 20 m sprint, and jump performance had statistical significance between elite and non-elite groups, including different positions and levels.

**Conclusions:**

The reviewed literature highlighted a complicated relationship between anthropometric, physiological, and physical performance. Based on the results of the study, it's possible that height, body mass, agility, speed, endurance capacity, and lower lamb power could affect the early development of basketball.

## 1. Introduction

Talent identification (TID) and development (TDE) are essential factors for elite sports' success (1, 2). The formalisation and standardisation of TID and TDE are highlighted in the research conducted by Christian and Lars (3), which also confirms the influence of multiple factors on talent development. Similarly, researchers have emphasised the significance of TID in the transition of athletes from the primary level to the elite level, as it helps them maintain a high level of performance and improves the athlete's overall environment (4, 5). Recent years have seen the development of structured TID and TDE programmes for several sports in which success is dependent on anthropometric, physiological, and motor skill attributes (6–8).

The phenomenological approach divides talents into raw materials and the culmination of their development. On the one hand, it refers to natural abilities that are innate, untrained, and spontaneous (aptitudes or talents) (9). On the other hand, early TID contributes to the success of athletes at the youth stage (10). Although a large sample of athletes demonstrated that the majority of children who were identified and supported early did not become elite athletes as adults (11), the majority of children who were identified and supported early did not become elite athletes as adults. It is impossible to ignore the effect of the athlete's developmental process on the athlete's catalysts (9). Individual talent development is commonly regarded as a business (12). Thus, a gifted individual is chosen for long-term development and nurturing (11). The Model of Giftedness and Talent (MDGT) includes an athlete's talent, physical performance, raw form, and acquired effort (4). Simultaneously, the biological context of talent identification suggests that anthropometrics, physiology, and physical performance are the most intuitive manifestations of what scouts look for in players (13) and are the most visible means of measuring the basis for the emergence of recruitment and selection programmes for the search, identification, and development of talent; physical fitness and physical ability determine, on the one hand, the factors necessary for an athlete to perform at a high level (14).

Basketball is a sport characterised by intermittent, high-intensity play, and optimal performance is achieved through a complex combination of technical and tactical skills (14), requiring players to perform a variety of intense defensive and offensive actions (sprinting, shuffling, jumping, etc.) and includes analysis of many physical and physiological attributes such as (height, weight, size, body proportions, aerobic capacity, strength, anaerobic capacity, agility, and speed) (14, 15). Height and body mass are essential for improving physical performance (16). Due to the layout of the basketball court, players of different heights occupy different positions, with shorter players moving the ball quickly around the court and larger, stronger, and higher players using their height and weight close to the basket to make effective shots (15, 17). Indeed, the anthropometric profile of an athlete is a significant predictor of his or her ability to compete at the highest level of his or her chosen sport (18).

Constant environmental and situational changes are a distinguishing feature of team sports (19–21) and athletic talent components are typically comparable across team sports. Moreover, physical fitness and physical performance are essential factors in determining basketball success (7). In addition to athletic expertise, the performance of players depends on a combination of physical, functional, and behavioural characteristics (22). On the one hand, the physical characteristics of players with different skill levels fluctuate (19). Conversely, basketball players may not need to excel in any particular area, but the vast majority are competent in all areas, especially those with higher skill levels who are faster, more agile, and perform better in vertical jump tests (23–25). In the studies that have been conducted on basketball selection, including anthropometric measurements and athletic and physiological performance of basketball players, there has been a consensus on how the different factors affect the selection process. This is true even though the different factors are affected by maturity, and more research needs to be done on the validity of the different aspects of selection that are tier one indicators.

Based on the above considerations, the purpose of this research was to conduct a systematic review and meta-analysis of anthropometric, physiological, and physical performance measures that best discriminate physical talent in basketball.

## 2. Materials and methods

### 2.1. Search strategy

This systematic review of the available literature was conducted according to the preferred reporting items for systematic review and meta-analysis protocols (PRISMA) 2020 guidelines (26).

**Figure 1:**
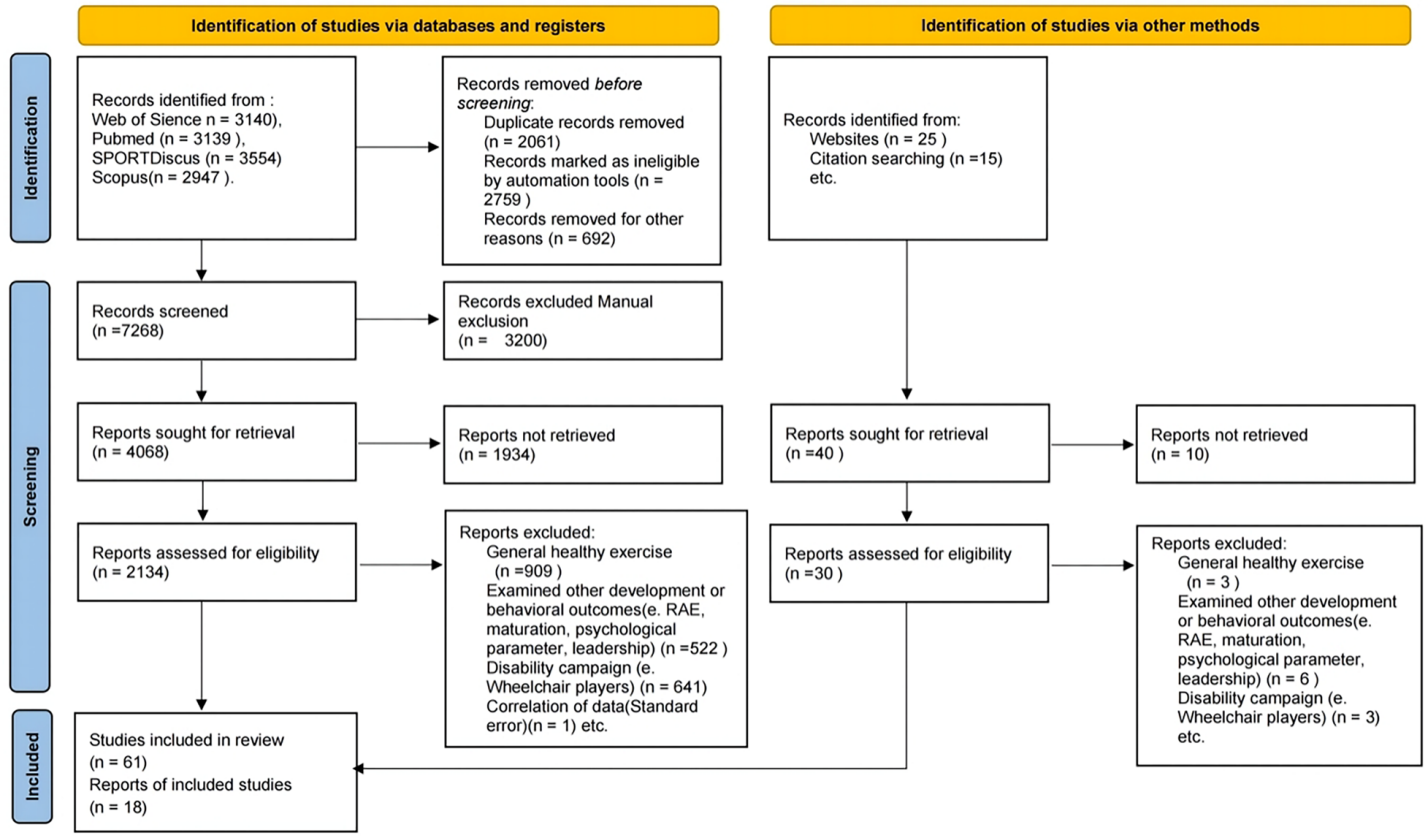
Flow diagram displaying the search's work flow.

Compiling scientific studies were carried out through an exhaustive and systematic search in three electronic databases: the Web of Science Core Collection, PubMed, SPORTDiscus and Scopus. The search terms used were grouped into four search strings: (1) “basketball” OR “basketballs” OR “basketballers” AND (2) “talent” OR “talents” OR “talented” AND (3) “identification” OR “identifications” AND (4) “anthropometric” OR “anthropometrical” OR “anthropometrically” OR “anthropometrics” AND (5) “physiologies” OR “physiology” AND (6) “physical performance” OR “performance” OR “physical functional performance”. The search process (Figure 1) was carried out in March 2020 and June 2022. The electronic search was supplemented by manually searching the reference lists for articles. A total of 12,780 articles from the database were searched using keywords. Duplicate and irrelevant articles were deleted. After reviewing the titles and abstracts, 79 records were eliminated, and through a comprehensive evaluation, eighteen quantitative articles were left.

### 2.2. Inclusion and exclusion criteria

To carry out this review, after the search for the articles, the study authors, titles, and dates of publication were recorded and the articles were sorted to eliminate duplicates. From the list of unique entries, the titles of each publication were examined to determine if they were written in English and consisted of a comprehensive peer-reviewed journal study. According to the PICOS question model, the inclusion criteria were: (1) population: players over the age of U12 years; (2) intervention: local/regional, gender, and athletic level; (3) comparison: association between individual and/or collective competition performance and player's fundamental movement skill, physiological parameters, and anthropometric parameters; (4) results: according to two specific indicators: level (individual and/or collective) and environment (different countries); and (5) study design: no specific limitations were set for study design.

Other examined developmental or behavioural results were excluded (e.g., RAE, maturation, psychological parameter, leadership), Disability campaign (e. Wheelchair players), Correlation of data (Standard error) etc. Excluded studies were published in the format of editorials, letters to the editor, comments, abstracts, conferences, or opinion articles.

### 2.3. Data collection and extraction

The review topics are related to the identification of basketball talent in various nations, taking into account comparisons between elite and non-elite basketball players and the age of the participants.

### 2.4. Quality of the studies

The organization of all article titles, authors, years, and source titles was explored in an Excel file. Two researchers independently conducted the search and screened the titles, abstracts, and full texts of the papers. The overall methodological quality of the studies was assessed using the McMaster's Critical Review Form: Quantitative studies (27). These studies were assessed to determine whether they included the following 16 items: objective (item 1), relevance of background literature (item 2), appropriateness of the study design (item 3), sample included (items 4 and 5), informed consent procedure (item 6), outcome measures (item 7), validity of measures (item 8), intervention (item 9), significance of results (item 10), analysis (item 11), clinical importance (item 12), description of drop outs (item 13), conclusion (item 14), practical implications (item 15), and limitations (item 16). Each of the articles' outcomes per item was designated as “1” (meets criteria) or “0” (does not meet the criteria fully).

Table 1 shows the study quality rating assessments: eighteen quantitative research articles. Eleven articles were deemed “very good” and seven articles were deemed “good.”

**Table 1:** The overall methodological quality for quantitative studies.

Author	1	2	3	4	5	6	7	8	9	10	11	12	13	14	15	16	TS	%	MQ
Arede et al. (28)	1	1	1	1	1	0	1	1	1	1	1	0	0	1	1	1	12	75%	G
Attene et al. (29)	1	1	1	1	1	1	1	1	1	1	0	1	0	0	1	1	13	81%	G
Guimarães et al. (41)	1	1	1	1	1	1	1	1	1	1	1	1	0	1	1	1	15	94%	VG
Fort-Vanmeerhaeghe et al. (30)	1	1	1	1	1	1	1	1	1	1	1	0	0	1	1	1	14	88%	G
Vernillo et al. (42)	1	1	1	1	1	1	1	1	1	1	1	1	0	1	1	1	15	94%	VG
Gryko et al. (31)	1	1	1	1	1	0	1	1	1	1	1	1	0	1	1	1	13	81%	G
Pojskic et al. (43)	1	1	1	1	1	1	1	1	1	1	1	1	0	0	0	1	13	81%	G
Bouteraa et al. (44)	1	1	1	1	1	1	1	1	1	1	1	1	0	1	1	1	15	94%	VG
Pino-Ortega et al. (45)	1	1	1	1	1	1	1	1	1	1	1	1	0	1	1	1	15	94%	VG
Ivanović et al. (46)	1	1	1	1	1	1	1	1	1	1	1	1	0	1	0	1	14	88%	VG
Torres-Unda et al. (25)	1	1	1	1	1	1	1	1	1	1	1	1	0	1	1	1	15	94%	VG
Masanovic et al. (32)	1	1	1	1	1	1	1	1	1	1	1	1	0	1	0	1	14	88%	VG
Natascia et al. (24)	1	1	1	1	1	1	1	1	1	1	1	1	0	0	1	1	14	88%	VG
Nikolaidis et al. (47)	1	1	1	1	1	1	1	1	1	1	1	1	0	0	0	1	13	81%	G
Pantelis Nikolaidis et al. (33)	1	1	1	1	1	1	1	1	1	1	1	1	0	1	1	1	15	94%	VG
Sérgio Antunes Ramos et al. (34)	1	1	1	1	1	1	1	1	1	0	1	1	0	1	1	1	14	88%	VG
Sérgio Ramos et al. (35)	1	1	1	1	1	1	1	1	1	0	1	1	0	1	0	1	13	81%	G
te Wierike et al. (36)	1	1	1	1	1	1	1	1	1	1	1	1	0	1	1	1	14	88%	VG

Ts, total items fulfilled by study; 1, criterion met; 0, criterion not met; MQ, methodological quality; P, poor ≤ 8 points; A, acceptable 9–11 points; G, good 12–13 points; VG, very good 14–16 points.

### 2.5. Description of data correlation and variability

To avoid using correlated data extracted from each study, Pearson's correlation analysis was performed on eight variables: height, body mass, Vo2 max, yo-yo test, *T*-test, squat jump (SJ), countermovement jump (CMJ), and 20-meter sprint. The means were analysed for variability using one-way ANOVAs.

The analyses were conducted using version 25.0 of the statistical software IBM SPSS for Windows (IBM Corp., Armonk, NY). Height, body mass, CMJ, and 20 m sprint correlation coefficients and the *T*-test were statistically significant with age; SJ was statistically significant with 20 m sprint correlation coefficients and one-way ANOVAs.

Different levels of correlation analysis confirmed that height and weight are significantly correlated with player position, with the exception of SJ, which was not correlated. SPSS was used to compare the correlations of various data. Due to excessive tables and data, different levels of data are subjected to a separate SPSS Pearson test at different locations, and the data results are then summarised in Excel to produce Tables 2, 3.

**Table 2:** Description of the correlation of variable data.

		Pearson Correlation							
		Height	BM	Yo-Yo test	Vo2max	SJ	CMJ	Sprint	Ttest
Age		**.** **829** **	**.** **803** **	.b	0.778	0.175	**.** **613** *	−.767**	−0.406
Position	DP	**.** **632** **	**.** **604** **	.b	.b	−0.962	−0.455	0.964	0.933
	Height	1	**.** **939** **			−0.924	0.121	0.926	0.884
	BM	**.** **939** **	1	.b	.b	−0.974	0.123	0.975	0.949
	Yo-Yo test	.b	.b	.b	.b	.b	.b	.b	.b
	Vo2max	.b	.b	.b	.b	.b	.b	.b	.b
	SJ	−0.924	−0.974	.b	.b	1	0.991	−1.000**	−0.996
	CMJ	0.121	0.123	.b	.b	0.991	1	−0.992	−0.975
	Sprint	0.926	0.975	.b	.b	−1.000**	−0.992	1	0.995
	Ttest	0.884	0.949	.b	.b	−0.996	−0.975	0.995	1
Different Level		**.** **801** **	**.** **776** **	**.966** **	**1.000** **	0.993	**.** **933** **	**.** **842** **	.999*

BM, body mass; G, gender; DP, different position (guard, forward, center); SJ, squat jump; CMJ, counter movement jump.

* At 0.05 level (two-tailed), correlation is significant.

** Significant correlation at 0.01 level (two-tailed).

b. Calculations could not be performed because at least one variable is a constant.

**Table 3:** Description of data variability.

ANOVA						
		SS	DF	MS	F	P
Height	Between groups	922.053	2	461.027	6.005	**0.01**
	Within group	1382.007	18	76.778		
BM	Between groups	1565.112	2	782.556	5.272	**0.016**
	Within group	2672.097	18	148.45		
SJ	Between groups	10.03	2	5.015	.	.
	Within group	0	0	.		
CMJ	Between groups	42.077	2	21.038	1.309	0.317
	Within group	144.688	9	16.076		
Sprint	Between groups	0.02	2	0.01	.	.
	Within group	0	0	.		
Ttest	Between groups	0.53	2	0.265	.	.
	Within group	0	0	.		

BM, body mass; G, gender; DP, different position (guard, forward, center); SJ, squat jump; CMJ, counter movement jump; SS, sum of squares; DF, degrees of freedom; MS, mean square; *P* = Significance (*P* < 0.05 significant values are given in bold).

### 2.6. Meta-analysis

Initial screening revealed that these variables were mentioned often enough to warrant a meta-analysis. Analysis based on correlation. To reduce error, data with correlations and differences between groups were included in the meta-analysis. Cochrane Collaboration's Review Manager 5.4.1, an open-source programme, was used to conduct the meta-analysis. For each study, the mean and standard deviation (SD) for each variable were used to figure out the 95% confidence interval (CI). In addition, meta-analyses with the same continuous outcome variable, unit of measurement, and meta-analyses with different units were conducted using the standardised mean difference (SMD). In each meta-analysis, the statistical significance of each study was determined using the inverse variance method, and either the random effects model (heterogeneity, *I*^2^ > 50%, *P* < 0.05) or the fixed effects model (heterogeneity, *I*^2 ^> 50%, *P* 0.05) was used to analyse the data (37). The only piece of information provided by confidence intervals is the standard deviation of the average timing.

In contrast, prediction intervals can predict the range of future samples by accounting for the variance between studies (the intervals are equal when *T*^2^ = *I*^2^ = 0). *I*^2^ was reported as heterogeneity between studies, reflecting the overlap between the CIs of several studies (37). *I*², which is calculated as follows:

$$I^{2}=[Q-(k-1)]/Q\times 100\%$$

Q is the chi-squared value of the heterogeneity test X² and k is the number of studies included in the meta-analysis. The Q statistic is the standardised weighted sum of squares of variance across studies, with lower *P*-values (typically around 0.010) indicating the presence of heterogeneity. The Q statistic usually has high statistical power when the number of included studies is large. The *I*^2^ statistic is the proportion of observed between-study variance due to true heterogeneity rather than that observed by chance. Since all values of *I*^2^ are taken as 0 when *I*^2^ is negative, *I*^2^ is taken between 0% and 100%. 0% *I*^2^ means that no heterogeneity is observed, and larger values indicate greater heterogeneity. The threshold for determining statistical significance of a mean difference was set at *P* < 0.05 (38). To determine whether the degree of similarity between the observed mean differences was statistically significant, the Q statistic was calculated. The Q statistic was then converted into a standard measure of homogeneity (*I*^2^ statistic) and used to evaluate the sample's level of heterogeneity. All performance variables included in the meta-analysis were subjected to sensitivity analyses to examine the robustness of the magnitude and direction of the performance effects of frequently mentioned variables. The sensitivity analyses were conducted by repeating the meta-analysis with the exclusion of studies with smaller sample sizes or unexpected results (39).

One of the articles used the mean and standard error, and the article was excluded to avoid bias (23, 40). Eighteen articles were eventually considered for inclusion in the review and meta-analysis. The main reason for the omission of an article was that it focused only on anthropomorphic characteristics and not on age or motor performance. Articles containing data from separate subgroups (age, level) were entered separately into the meta-analysis if they met the inclusion criteria. As these were natural basketball conditions, all data from studies with multiple condition/age points were used in the meta-analysis. Multiple condition/age points were combined into a single estimate for each study as these data were not independent and would have led to an overestimation of the precision of the meta-analysis.

## 3. Results

There are eighteen articles listed in Table 4, and 74% (*n* = 13) of the studies examined male only samples. Only 16% (*n* = 3) of the studies examined a female-only sample, while the remaining 10% (*n* = 2) used male and female participants. The only sport represented was basketball. Few studies have been conducted on basketball. Most studies looked at how a player's body size, physiology, physical performance, and performance affect his or her talent and competitiveness in basketball Table 4. The Summary of Anthropometric, Physiological, and Physical Performance Factors in Team Sports principal test and results.

**Table 4:** The summary of anthropometric, physiological, and physical performance factors in team sports principal test and results.

Author	Sample	G	Age	Character	Anthropometric		Physiology		Physical Performance				Main results
					Height (cm)	Body mass (kg)	Yo-Yo test (m)	VO2max (ml/kg/min)	SJ (cm)	CMJ (cm)	20 m Sprint (s)	*T*-Test (s)	
Arede et al. (28)	Players on the national team	M	U13 (*n* = 36)	MS	160.26 ± 10.71	49.37 ± 13.71			24.27 ± 4.83	24.72 ± 3.36	3.77 ± 0.22		M: Height, body mass, SJ, CMJ, and 20 m: LS > MS
				LS	169.36 ± 13.69	56.4 ± 13.6			26.22 ± 4.44	26.07 ± 3.59	3.72 ± 0.15		
		F	U13 (*n* = 32)	MS	162.34 ± 6.83	58.58 ± 12.68	–	–	20.65 ± 3.53	20.09 ± 3.21	3.99 ± 0.21	–	
				LS	162.05 ± 9.98	56.59 ± 7.79			24.4 ± 3.26	23.75 ± 2.99	3.85 ± 0.16		
Attene et al. (29)	Players on the national team	F	U15 (*n* = 16)	IT	164 ± 4	53.50 ± 4.63	605 ± 233						Height, body mass, and Yo-Yo test: RST > IT
				RST	167 ± 8	55.30 ± 3.16	720 ± 291						
Guimarães et al. (41)	Players on the reginal team	M	U14 (*n* = 150)	E	177.4 ± 6.2	66.1 ± 8.2	1097.5 ± 411.6		26.3 ± 5.1	29.3 ± 7.2	3.3 ± 0.2	9.2 ± 0.4	Height, body mass, Yo-Yo test, SJ, CMJ, 20 m, *T*-test: E > NE
				NE	163.1 ± 10.0	52.8 ± 10.7	714.6 ± 322.7		25.1 ± 6.2	30.0 ± 5.8	3.7 ± 0.3	10.0 ± 0.6	
Fort-Vanmeerhaeghe et al. (30)	Players, play on the national team	F	U16 (*n* = 9)		180 ± 0.08	72.3 ± 14.3		45.90 ± 2.61	21 ± 3	24 ± 5		11.04 ± 0.66	Heigh, body mass, VO2max, *T*-Test, and CMJ: U18 > U16.
			U18 (*n* = 11)		182 ± 0.07	70.17 ± 8.18		46.59 ± 1.81	24 ± 2	27 ± 3		10.8 ± 0.51	
Vernillo et al. (42)	Players on the national and reginal team.	M	U14 (*n* = 30)	E NE E NE	168.6 ± 10.9 167.5 ± 8.1	59.8 ± 14.9 58.7 ± 10.1	1110 ± 385 729 ± 345						Height, body mass, and Yo-Yo test: E > NE
			U15 (*n* = 29)		174.5 ± 7.8 169.9 ± 10.3	66.4 ± 13.8 60.9 ± 13.8	1283 ± 461 796 ± 289						
			U17 (*n* = 29)	E NE	186.1 ± 4.7 181.9 ± 7.1	79.5 ± 9.1 73.7 ± 10.5	1412 ± 245 1078 ± 565						
Gryko et al. (31)	Players, play in national and regional team	M	U14 (*n* = 35)	Guard	169.36 ± 5.16	57.10 ± 6.64							U14, height and body mass: centres > forwards > guards.
				Forward	182.59 ± 3.81	63.71 ± 6.67							
				Center	185.98 ± 3.39	74.02 ± 10.51							
			Adult (*n* = 35)	Guard	186. 68 ± 5.9	81.08 ± 4.61							Adult, height, and body mass: centres > forwards > guards.
				Forward	193.85 ± 4.39	89.25 ± 8.55							
				Center	199.83 ± 7.37	100.29 ± 7.10							
Pojskic et al. (43)	Players in national team		Adult (*n* = 38)		185.35 ± 6.73	78.66 ± 10.35		63.67 ± 6.79	31.09 ± 0.09	38.48 ± 3.45	3.14 ± 0.09	10.48 ± 0.409	All the results are statistically significant.
Bouteraa et al. (44)	Players in national and reginal team.	F	U17 (*n* = 26)	E	168 ± 5	56.6 ± 8.3			20.4 ± 3.9	26.8 ± 3.8	3.39 ± 0.25		Height, body mass, 20 m, SJ, and CMJ: E > C.
				NE	168 ± 8	55.6 ± 7.0			20.4 ± 2.5	25.2 ± 2.9	3.46 ± 0.16		
Pino-Ortega et al. (45)	Players in national and reginal team.	F	U16 (*n* = 42)	E NE	174.4 ± 6.3 170.4 ± 6.8	64.0 ± 7.6 64.7 ± 8.7	286 ± 75 265 ± 60			43.8 ± 5.9 40.9 ± 5.4	3.74 ± 0.18 3.85 ± 0.13		Heigh, body mass, Yo-Yo test, 20 m and CMJ: U18 > U16.
			U18 (*n* = 29)	E NE	178.9 ± 5.3 175.5 ± 5.1	69.3 ± 5.6 68.5 ± 9.8	340 ± 106 283 ± 95			48.9 ± 6.1 43.3 ± 5.2	3.69 ± 0.18 3.86 ± 0.20		
		M	U16 (*n* = 37)	E NE	183.1 ± 8.2 184.4 ± 6.2	72.5 ± 12.6 74.8 ± 9.5	443 ± 85 410 ± 109			56.1 ± 10.6 52.8 ± 7.9	3.49 ± 0.16 3.53 ± 0.15		
			U18 (*n* = 30)	E NE	188.3 ± 5.9 187.3 ± 6.2	76.8 ± 9.8 77.8 ± 8.3	465 ± 139 477 ± 190			57.2 ± 10.7 55.9 ± 8.0	3.40 ± 0.12 3.41 ± 0.14		
Ivanović et al. (46)	Players in national team	M	U17 + U19 (*n* = 61)	Guard	192.80 ± 4.49	79.83 ± 6.94			34.59 ± 5.77	41.16 ± 6.20	3.002 ± 0.117	10.321 ± 0.402	Height, body mass, SJ, CMJ, 20 m: Centres > Forwards > Guards.
				Forward	201.48 ± 3.14	90.93 ± 9.85			33.49 ± 5.70	39.15 ± 5.93	3.052 ± 0.180	10.481 ± 0.711	
				Center	207.20 ± 3.29	104.00 ± 9.64			30.28 ± 4.69	35.79 ± 4.33	3.194 ± 0.128	11.282 ± 0.695	
Torres-Unda et al. (25)	Players in national and reginal team.	M	U13 (*n* = 62)	E NE	180.55 + 6.86 168.79 + 9.89	70.33 + 13.69 57.34 + 8.71				46.75 + 6.075 33.373 + 7.196	3.02 + 0.27 3.28 + 0.35		Height, body mass, 20 m, and CMJ: E > NE.
Masanovic et al. (32)	Players in national and reginal team	M	U18 (*n* = 33)		193.60 ± 7.70	80.00 ± 9.76							All the results are statistically significant.
Rinaldo et al. (24)	Players in reginal team	M	U13 (*n* = 50)		155.22 ± 9.17	47.62 ± 11.77			23.88 ± 3.72	25.59 ± 3.79	3.35 ± 0.27	11.95 ± 1.17	Post result > pre
Nikolaidis et al. (33)	Players in first academy club. (*n* = 72)	M	U12 (*n* = 32) U15 (*n* = 23) U18 (*n* = 17)		145.8 ± 8.7 168.1 ± 10.0 182.4 ± 6.2	40.0 ± 8.4 61.5 ± 11.3 80.3 ± 11.0				23.9 ± 5.6 31.8 ± 6.1 38.6 ± 7.0	4.07 ± 0.37 3.67 ± 0.27 3.27 ± 0.11		Height, Body mass 20 m, CMJ, U18 > U15 > U12
Nikolaidis PT et al. (47)	Players on the national team.		U15 (*n* = 35) U18 (*n* = 35) E (*n* = 31)	Guard Forward Center Guard Forward Center Guard Forward Center	172.3 ± 6.0 180.4 ± 7.6 190.1 ± 4.3 183.8 ± 7.4 186.8 ± 8.1 190.4 ± 4.6 186.7 ± 7.5 197.0 ± 3.9 207.6 ± 4.6	68.1 ± 9.3 77.4 ± 11.7 82.4 ± 7.4 76.2 ± 9.8 78.1 ± 11.6 89.8 ± 18.0 87.2 ± 10.5 95.1 ± 5.8 109.4 ± 12.4				36.4 ± 9.2 33.2 ± 6.6 34.8 ± 2.5 41.9 ± 5.9 41.1 ± 4.2 37.4 ± 5.8 45.1 ± 3.3 46.9 ± 7.8 39.0 ± 4.3			Height, body mass, 20m: Centres > Forwards > Guards.
Ramos et al. (35)	Players in reginal team	M	U14 (*n* = 55) U16 (*n* = 61)	TA TB TA TB	167.50 ± 11.27 158.30 ± 11.29 186.13 ± 9.73 172.73 ± 11.51	53.89 ± 12.50 49.32 ± 15.10 72.21 ± 11.44 59.83 ± 13.04		44.07 ± 2.33 42.10 ± 2.18 47.27 ± 4.74 47.44 ± 3.67	25.84 ± 4.64 24.33 ± 5.32 30.35 ± 4.97 27.89 ± 4.98	26.54 ± 5.25 25.14 ± 6.12 31.87 ± 5.12 29.51 ± 5.25	3.55 ± 0.24 3.70 ± 0.31 3.28 ± 0.18 3.32 ± 0.17	11.47 ± 0.73 12.37 ± 0.98 10.17 ± 0.57 10.91 ± 0.79	Height, body mass, Vo2max, 20 m, SJ, CMJ, *T*-test: TA > TB.
Ramos SA et al. (34)	Players in best Club and reginal team.	M	U14 (*n* = 173) U16 (*n* = 108)		173.5 ± 8.4 180.5 ± 7.31	60.1 ± 9.9 68.8 ± 9.8				30.4 ± 4.8 34.4 ± 4.6	3.35 ± 0.22 3.12 ± 0.11	10.35 ± 0.60 9.55 ± 0.50	Height, body mass, 20 m, SJ, CMJ, *T*-test: U16 > U14
te Wierike et al. (36)	Players, play in national team	M	U15 (*n* = 43)	Guard	170 ± 10	54.39 ± 10.56							Height and body mass: Centres > Forwards > Guards.
				Forward	183 ± 8	69.95 ± 13.09							
				Center	183 ± 15	71.78 ± 14.14							

G, gender; M, male; F, female; SJ, squat jump; CMJ, counter movement jump; MS, much specialization; LS, less specialization; IT, intermittent training; RST, repeated sprint ability training; E, elite; NE, non-elite; E, experimental group; C, control group; TA, team A = Elite players; TB, team B = non-elite players.

### 3.1. Anthropometric

**Figure 2:**
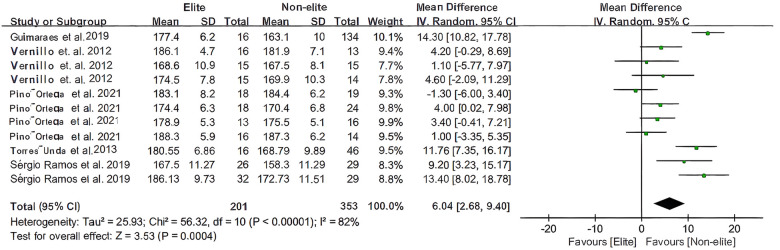
The youth players’ height meta-analysis.

**Figure 3:**
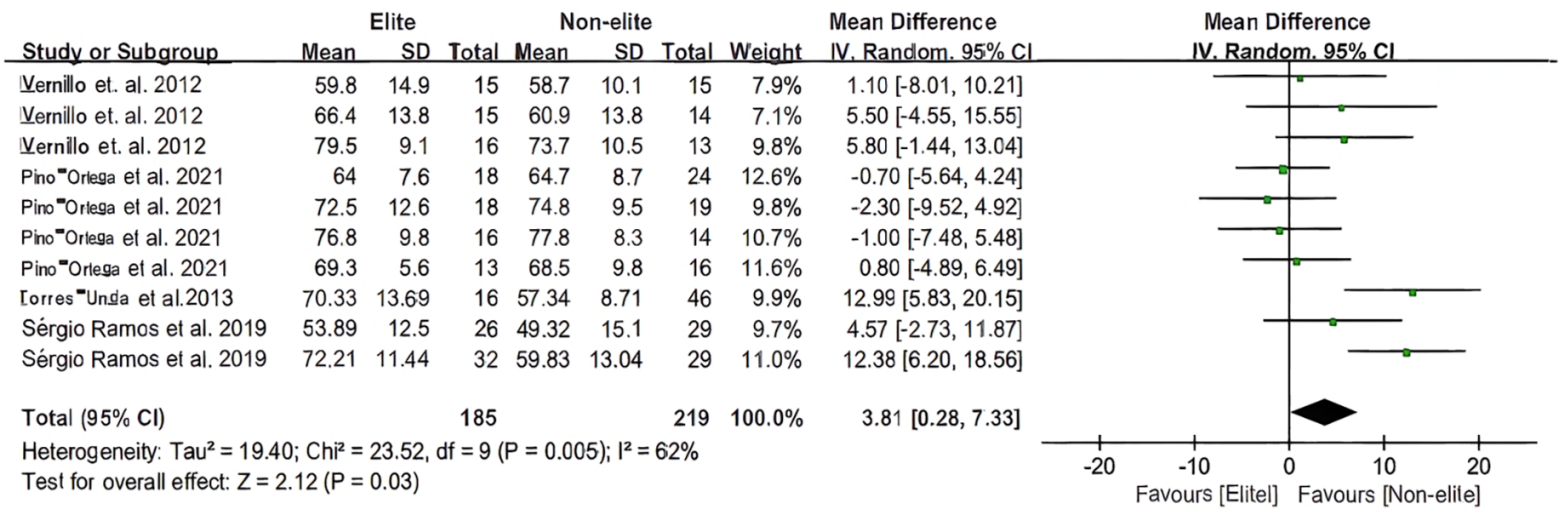
The youth players’ body mass meta-analysis.

The total samples (*n* = 11; including male and female samples of varying ages). In height and body mass, due to the different data (mean and SD) in the article, this study compared the heterogeneity of height and body mass of basketball players in the article based on different levels, as shown in Figures 2, 3 (Hedges g (MD) = 6.04, 3.81; 95% CI [2.68 to 9.40], [0.28 to7.33]; *P* < 0.00001, *P* = 0.005). The total test for overall effect as an effect test result *Z* = 3.53, *P* = 0.0004 < 0.05; *Z* = 2.12, *P* = 0.03 < 0.05 indicate that the data pertaining to the mobilisation of basketball players of different age groups combined are statistically significant, irrespective of the height and body mass of players at different levels.

### 3.2. Physiology

**Figure 4:**
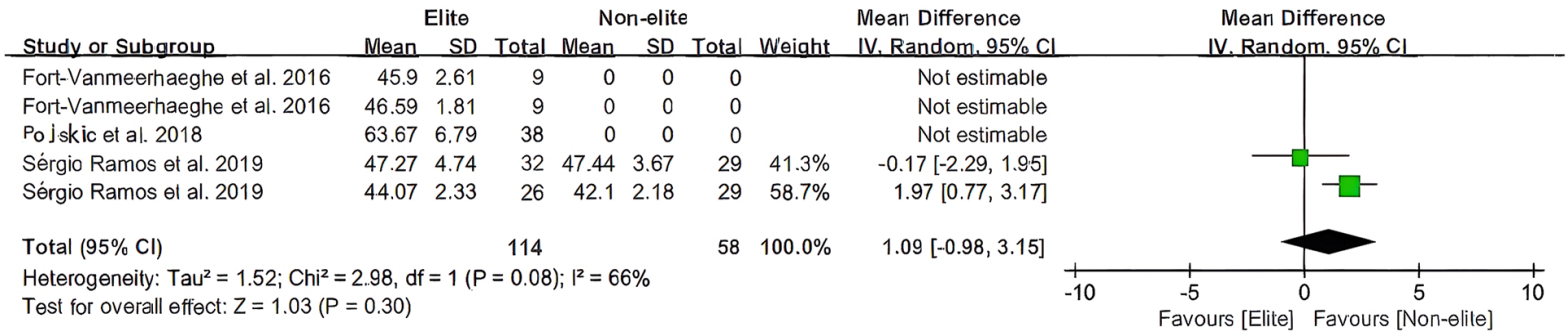
The youth player Vo2max meta-analysis.

**Figure 5:**
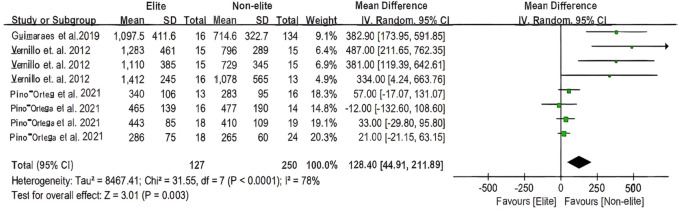
The youth player Yo-Yo test meta-analysis.

Figures 4, 5 for the forest plot of Vo2 max and the Yo-Yo test meta-analysis, respectively. No low-level group was available for the excluded data. The overall heterogeneity of samples (*n* = 2, *n* = 8) was moderately high (*I*^2^ = 66%, *I*^2^ = 78%). The overall mean was estimated to be 1.09 milliseconds ahead of the total [95% CI: (−1.98 to 3.15)ms] and 128.4 milliseconds ahead of the total [95% CI: (44.91 to 211.89) ms]. In basketball, there is no statistical difference in the overall effect of the Vo2 max test (*Z* = 1.03, *P* = 0.30 > 0.05), whereas there is a statistical difference in the test for overall effect of the Yo-Yo test (*Z* = 3.01, *P* = 0.003 < 0.05).

### 3.3. Physical performance

**Figure 6:**
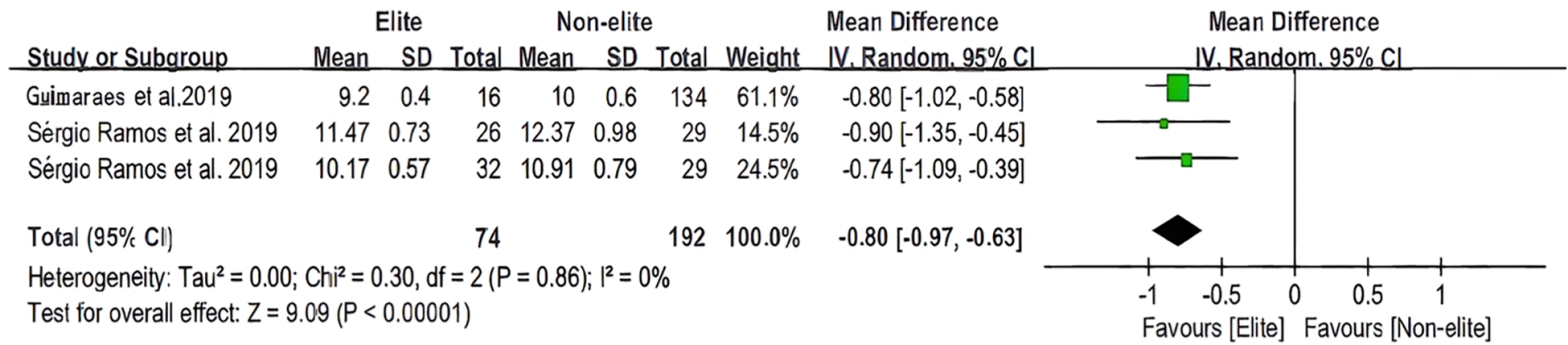
The youth player *T*-test meta-analysis.

**Figure 7:**
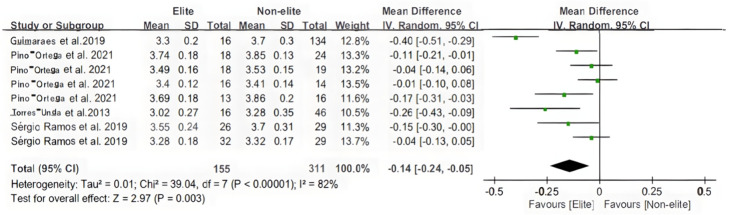
The youth player 20 m meta-analysis.

Figures 6, 7 (*T*-test and 20 m sprint) represent the outcomes of the identical analysis conducted on each variable for the elite and non-elite groups. Forest plots for the *T*-test and 20-m meta-analysis are depicted in the figures. In addition, the overall heterogeneity of the samples (*n* = 3, *n* = 8) was relatively high (*I*^2^ = 0%, *I*^2^ = 82%). The overall mean was estimated to be −0.80 milliseconds before the total [95% CI: (−0.97 to −0.63) milliseconds] and −0.14 milliseconds before the total [95% CI: (−0.24 to −0.05) ms]. In basketball, there is a statistically significant difference (*Z* = 9.09, *P* < 0.00001; *Z* = 2.97, *P* = 0.003 < 0.05) in the test for the overall effect of the *T* test and 20-meter sprint between the elite and non-elite groups.

**Figure 8:**
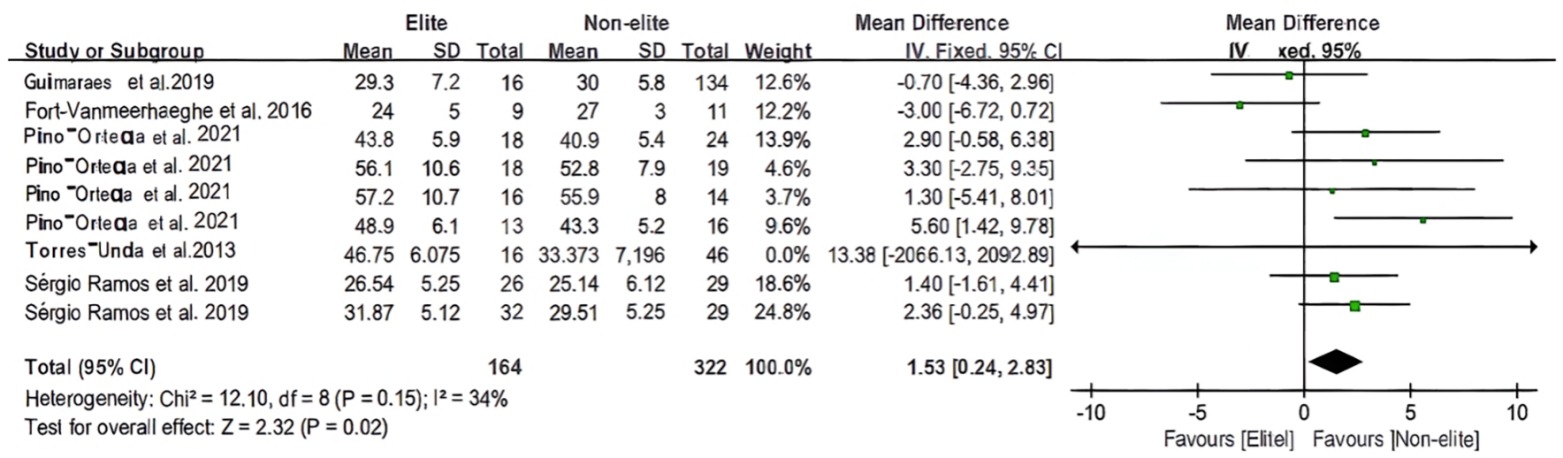
The youth player CMJ meta-analysis.

**Figure 9:**
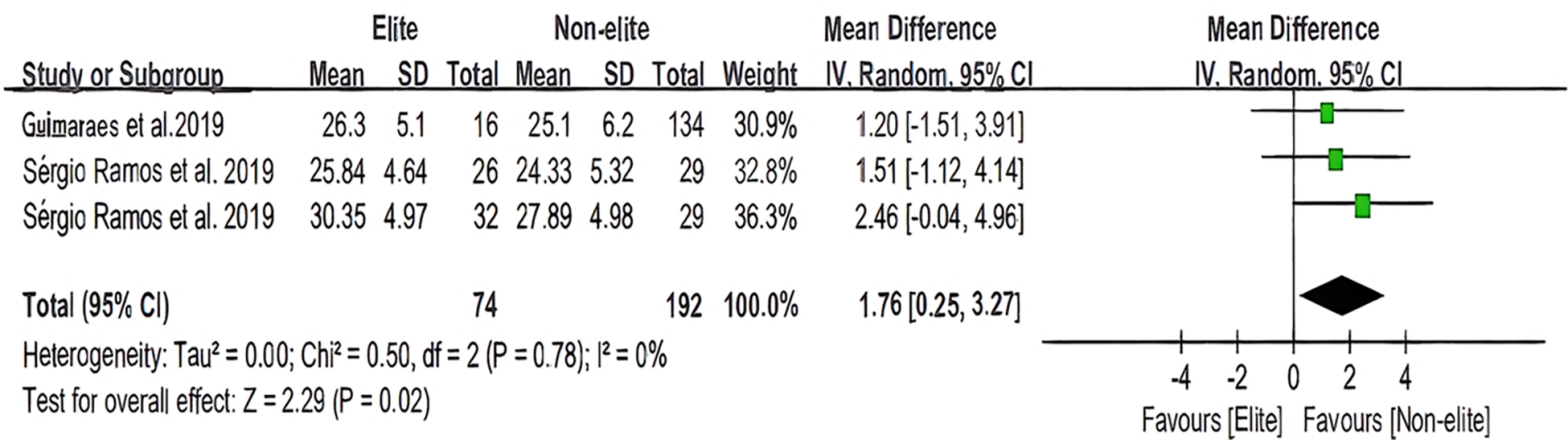
The youth player SJ meta-analysis.

Figures 8, 9 depict the statistical and forest plots of the CMJ and SJ meta-analyses separately for each age group. The heterogeneity of samples (*n* = 9, *n* = 3) was relatively high (*I*^2^ = 34%, *I*^2^ = 0%). The overall mean was estimated to be 1.53 ms before the total [95% CI: (0.24 to 2,83) ms] and 1.76 ms prior to the total [95% CI: (0.25 to 3.27) ms]. The test for overall effect indicated (*Z* = 2.32, *P* = 0.02 < 0.05; *Z* = 2.29, *P* = 0.02 < 0.05) that the heterogeneity of total samples was significant in both the CMJ and SJ data analyses. Therefore, there is a statistical difference in the performance of basketball players on the CMJ and SJ tests.

## 4. Discussion

The purpose of this systematic review and meta-analysis was to identify factors of anthropometric, physiological and physical performance factors in the selection of basketball. The significance of anthropometric characteristics and physical performance in identifying basketball players was confirmed by the research. However, unlike previous studies, Vo2max did not represent the number of factors associated with athlete selection in the meta-analysis, and both metrics (Yo-Yo test and Vo2max) included the aerobic and anaerobic capacity of the athletes; however, additional confirmation is required. By comparing commonly measured statistics (such as body composition, physical performance, and physiological parameters), no athletes were excluded from the analysis of this study.

### 4.1. Anthropometric

In this review, some articles (*n* = 7) show height and body mass differ in elite and non-elite players. For instance, basketball players are always selected at a young age based on their height and larger body size (25, 36, 47). Studies on elite teams, such as the NBA, indicate that height and weight vary as players ascend the rungs, with height and weight being significant factors in shooting and passing (14, 15, 17). Moreover, the selection process for youth basketball tends to apply to all playing positions, and coaches tend to select athletes who fit an elite basketball profile characterised by a tall stature (35).

Overall height and body mass are among the most intuitive and effective ways to identify athletes in basketball (15). Earlier research suggested that coaches would select athletes based on competition and overlook the possibility of many athletes becoming elites in the future (48). Through meta-analysis, this paper demonstrates that there is no overall heterogeneity in height and body mass across levels, age, and gender, and that height and body mass are statistically significant across both athletic level and positional differences.

### 4.2. Physiology

Based on the characteristics of basketball, aerobic and anaerobic capacity can be seen as a distinguishing characteristic in the selection of basketball players, particularly the Vo2 max and Yo-Yo tests, which are useful in team sports and are important in screening articles to measure the aerobic and anaerobic capacity of basketball players. Improve anaerobic exercise recovery during a game, for example, by repeating short sprints or high intensity acceleration recovery intervals and preparing players to maintain an appropriate training load (29, 42). Through meta-analysis, the present study determined that the results of using the yo-yo IR1 to differentiate aerobic performance between elite, sub-elite, and non-athletic groups were consistent with the findings of Vernillo et al. (42). This study also revealed that the best young basketball players possessed superior aerobic fitness (41). In addition, the Yo-Yo test has been validated as a basketball test because its physiological demands are comparable to those of the game. Consequently, training with a Yo-Yo can affect the anaerobic energy system, which is crucial for team sports such as basketball (29).

Aerobic endurance and anaerobic capacity performance varied by competitive level, with elite athletes performing better (49–52). Anaerobic capacity is related to changes in peak height velocity and body size (53). Basketball is a high-speed running sport in which anaerobic capacity and aerobic capacity can impact player performance (54). When played on the same court, basketball necessitates a great deal of space and positional changes (28, 29, 41). At this point, the Yo-Yo test demonstrates the aerobic capacity by highlighting the body's ability to rapidly change intensity and accelerate over short distances (29). And again, aerobic and anaerobic capacity were significantly associated with assists and interceptions (35). Nevertheless, their maximum oxygen uptake is strongly influenced by genetics (55), developmental stage, and age group stratification, and the effect of age on their basic physical fitness may be overlooked during early selection. Consider the fact that the meta-analysis of Vo2max in basketball players presented in this article is not statistical (34); it was determined in this study that maximal oxygen uptake did not influence selection. In Groky's study, it was determined that among the predictors, U13 has a poorer endurance result, and that pubertal development influences endurance (14, 23). In Pojskic's research, it was determined that aerobic capacity is not a factor in basketball shooting (43). This result was likely the result of the homogeneity of the aerobic power, which reduced the variance and correlation between this conditioning capacity and the shooting performance. Due to the nonlinear nature of aerobic and anaerobic capacities, special care must be taken when selecting based on index values obtained prior to puberty.

### 4.3. Physical performance

According to the characteristics of basketball, the importance of agility in team sports could be explained by the frequent occurrence of situations requiring multiple, rapid direction changes in a small court (30, 35). Recent research has also highlighted agility as an essential attribute for youth team sports (30, 56). For instance, U14 basketball players on higher-ranked teams have demonstrated greater agility than those on lower-ranked teams (56). It appears that the *T*-test is better suited for differentiating between players of various competitive levels and training backgrounds. Therefore, it should be considered as a possible method for comparing and selecting the best athletes (24, 34, 41). In light of the fact that the *T*-test is the most traditional of the literature screenings and employs a literature perspective, it is essential not to overlook the importance of the agility test in selecting basketball players. And, based on this paper's meta-analysis, its characteristics are significant in younger age groups.

Concerning speed and jump performance evaluations, it has been shown that elite athletes perform better than their peers on speed and lower limb power tests (28). SJ, CMJ are a significant factor when evaluating an athlete's lower limb strength, particularly in basketball, according to the majority of the literature included in the meta-analysis. For the Caps to win the game, exceptional jumping ability in shooting and passing may also be crucial (24, 28, 33, 41). Its ability to leap not only reflects the muscular strength of its lower limbs, but also plays a vital role in its explosive power. Although its performance on the field requires a combination of physical qualities, the selection of fundamental physical qualities is essential for the long-term development (50, 51). According to the study (50, 57) the elite players also performed better on the shuttle run, variable-distance sprinting, and dribbling tests. And this study revealed that jumping is statistically significant for both elite and non-elite athletes, making it an unavoidable factor in early selection and the gradual development of speed qualities.

It is reported that athletes who are able to jump higher could have an advantage over their adversaries during offensive game actions (47). For instance, basketball players should jump as high and fast as possible to find better opportunities to shoot (47, 58). Superior dynamic vertical jump qualities may allow players to positively engage in aerial ball contests (i.e., when the ball is kicked to a group of players) during gameplay (59). Probably, the selection of players more prone to competing at the highest levels of human performance (i.e., Olympic team athletes) relies more on physical traits directly associated with the majority of tasks performed throughout the games (22). Overall, in long-term development, this can reflect the sports conditions. Even though using a single measurement can lead to mistakes in judgment, the multidimensional combination of physical changes, necessary athletic skills, congenital conditions, and learned sports factors is better for long-term development.

## 5. Limitation

As noted above, the variables considered omitted perceptual-cognitive, tactical, and psychological characteristics. These data were not included because research on youth players’ perceptual-cognitive skills is quite limited and generally laboratory-based (52, 60). These factors are not easy to consider in TID in many countries, especially in terms of the overall development of athletes.

Furthermore, in this study, basketball is considered a physically demanding sport in which strength and power play an important role. As physical maturity is associated with muscular strength, endurance, and speed and is important to achieve successful performance, relatively older athletes may be preferred over younger ones (61). The fact that even relatively young athletes can compete at a senior level, i.e., the effect of RAE on the long-term development of athletes, was not taken into account in this study and needs to be further investigated. In addition to this, maturity has a significant influence on height, weight, and athletic performance in the selection of athletes at the youth level (62, 63), the results resulting from the influence of maturity on athletes were excluded from the data selection in this article. This study shows significant differences between elite and non-elite athletes in functional ability and sport-specific skills comparing multiple countries using a standard method (52, 63). Both maturity and psychological factors must be analysed in more profound research in the next studies.

## 6. Conclusions

The reviewed literature highlighted a complicated relationship between anthropometric, physiological, and physical performance. Based on the results of the study, it is possible that height, body mass, agility, speed, endurance capacity, and lower body power could affect the early development of basketball. Particularly in the early stages of basketball development, certain factors can influence a young player's ability to learn basketball and excel in the game. Children who are taller and more physically developed may initially have an advantage due to their ability to reach the basket and cover more area on the court. Although children's physical abilities may not be fully developed, early exposure to activities that build endurance and lower body strength can set the stage for better performance in the future. Activities that promote cardiovascular fitness and muscle strength contribute to athletic performance. Agility and speed are skills that can be developed from an early age through a variety of exercises and drills. Early development of these skills can give players an advantage in manoeuvring on the court, defending, and executing offensive plays. For young players, early development in basketball involves a combination of talent and skill acquisition. Although certain physical attributes may provide an initial advantage, skill development, practise, and instruction are critical to maximising potential.

## 7. Practical applications

The review highlights the complex relationship between anthropometry, physiology, and physical performance when identifying talent among basketball players and the implications for early identification of athletes. Coaches and scouts select athletes while also considering how the athlete will perform in the game.

In the meta-analysis, Vo2max did not represent the number of factors associated with athlete selection, and both metrics (yo-yo test and Vo2max) included aerobic and anaerobic capacity; further confirmation is needed to determine whether it is affected by RAE as well as maturity (the role of athlete strength and power in the game).

The results of this study suggest that height, weight, yo-yo test, *T* test, 20 m sprint, and jumping scores are statistically significant between elite and non-elite groups in the talent selection of youth basketball players, that early athlete identification can be achieved through objectively measurable factors, and that the long-term development of athletes should be considered in a more multifaceted manner. As well as the need to consider the fairness of athletes' participation in the youth federation rules and championship rules, factors such as athletes' physical measurements can be appropriately added to the participation registration.

## Data Availability

The original contributions presented in the study are included in the article/Supplementary Material, further inquiries can be directed to the corresponding author.
